# Self-management interventions for chronic widespread pain including fibromyalgia: a systematic review and qualitative evidence synthesis

**DOI:** 10.1097/j.pain.0000000000003379

**Published:** 2024-09-17

**Authors:** Xiao-Yang Hu, Ben Young, Miriam Santer, Hazel Everitt, Jen Pearson, Hannah Bowers, Michael Moore, Paul Little, Tamar Pincus, Cathy Price, Tom Robson, Clara de Barros, Jane Loewy, Jenny Magee, Adam W. A. Geraghty

**Affiliations:** a Primary Care Research Centre, School of Primary Care, Population Sciences & Medical Education, Faculty of Medicine, University of Southampton, Southampton, United Kingdom; b School of Health and Wellbeing, University of Glasgow, Glasgow, United Kingdom; c School of Health and Social Wellbeing, University of the West of England, Bristol, United Kingdom; d The RNHRD and Brownsword Therapies Centre, Royal United Hospital, Bath, United Kingdom; e School of Psychology, University of Southampton, Southampton, United Kingdom; f Pain Clinic Solent NHS Trust, Southampton, United Kingdom; g Surrey and Borders Partnership NHST, Surrey, United Kingdom; h Public Contributor, Surrey, United Kingdom; i Public Contributor, Lichfield, United Kingdom; j Public Contributor, Winchester, United Kingdom

**Keywords:** Self-management, Self-care, Chronic widespread pain, Fibromyalgia, Systematic review, Qualitative

## Abstract

Supporting behavioural self-management is increasingly important in the care for chronic widespread pain (CWP), including fibromyalgia. Understanding peoples' experiences of these interventions may elucidate processes and mechanisms that lead to or hinder their intended impact. We conducted a systematic review and thematic synthesis of qualitative studies exploring peoples' experiences of self-management interventions for CWP, including fibromyalgia. MEDLINE, Embase, PsycINFO, CINAHL, and Web of Science were searched. Primary qualitative or mixed-methods studies were included if they explored people's self-management intervention experiences for their CWP, including fibromyalgia. Screening, data extraction, and critical appraisal were conducted by 2 reviewers. Data analysis was conducted through thematic synthesis. Twenty-three studies were included, mostly were rated as high or moderate quality. We developed 4 analytic themes: A multifaceted experience of the intervention, potential for transformative experience of group cohesion, a new outlook, and striving for change after the loss of support. Broadly, personalisation was perceived as beneficial and people experienced a range of emotional experiences. These appeared to support positive behavioural and cognitive changes. For most, group activities promoted acceptance and support, fostering new perspectives and improved self-management, although some found aspects of group contexts challenging. Lack of on-going support after interventions led to challenges in applying behavioural strategies, and some struggled without social support from the group. The experiences of self-management interventions for CWP reflect a complex, multifaceted process. Although many reported positive experiences, addressing issues with integration of physical activity, group dynamics and postintervention support may improve effectiveness for a broader range of people.

## Abstract 2

Supplemental Digital Content is Available in the Text.

## 1. Introduction

Chronic widespread pain (CWP) is diagnosed when persistent pain is reported over multiple body sites. It is a key feature of conditions, such as fibromyalgia, where it is accompanied by other symptoms, such as sleep disturbances, fatigue, and cognitive issues.^4^ The reported prevalence of CWP in the general population falls between 9.6% and 15%.^1,32^ Fibromyalgia, at the more severe end of the CWP spectrum, has a prevalence around 2% to 4%, with the highest prevalence in those aged between 40 and 60 years.^25^ In addition to pain, fatigue, disrupted sleep, difficulties with concentration, anxiety, and depression are also characteristics of fibromyalgia.^49^ The aetiology and pathogenesis of CWP remains unclear^27^; however, a genetic influence has been demonstrated,^27^ and central sensitisation, which refers to an abnormal amplification of neural signalling within the central nervous system, may be a primary mechanism.^7^ In addition, there is an association between chronic pain and life traumas, especially childhood trauma.^26^

Increasing emphasis is being placed on the need to support people with CWP to effectively engage with psychosocial and behavioural self-management: guidelines recommend a limited range of pharmacotherapies to treat severe symptoms,^30^ with a primary focus now placed on nonpharmacological interventions as first-line care.^30^ Self-management is a complex construct and has been defined as a patient-directed approach with the overarching aim of enhancing participants' health status or quality of life.^35^ Self-management approaches seek to bolster participants' skills and knowledge, enabling them to apply these enhanced abilities in various aspects of their lives beyond the intervention itself.^35^ These interventions often include multiple components, such as exercise and psychological elements, and are designed to improve outcomes directly.^35^

A systematic review of randomised controlled trials of self-management for CWP including fibromyalgia demonstrated small to moderate beneficial effects of self-management interventions on physical function and pain when compared with usual care in both the short and long term.^20^ Another systematic review showed similar findings of multicomponent interventions, including psychological and physical activities with short-term effects in people with fibromyalgia.^23^ However, there was considerable variability in the outcomes reported. To determine how this variance could be reduced and to improve the overall effectiveness of self-management approaches, there is a need for greater understanding of intervention processes and mechanisms that may drive or hinder benefit from these interventions.

Numerous primary qualitative studies have explored peoples' experiences of self-management interventions for CWP.^33,41,44^ In this study, we aimed to systematically review and synthesise their findings. We wished to explore common key processes that may be important in affecting the impact of self-management interventions for people experiencing CWP, including fibromyalgia.

## 2. Methods

### 2.1. Registration and protocol

An outline of this protocol was registered with the International Prospective Register of Systematic Reviews (PROSPERO CRD42022346261). This systematic review of qualitative studies is reported according to Preferred Reporting Items for Systematic Reviews and Meta-Analyses statement^29^ (PRISMA, Appendix A, http://links.lww.com/PAIN/C119) and the Enhancing Transparency in Reporting the Synthesis of Qualitative Research statement (ENTREQ, Appendix B, http://links.lww.com/PAIN/C119).^55^

### 2.2. Information sources and search strategy

MEDLINE (Ovid), Embase (Ovid), PsycINFO (EBSCOhost), CINAHL (EBSCOhost), and Web of Science were searched from inception to November 17, 2023. A supplemental reference list check and forward citation search^10^ of included studies were carried out. Search strategies were created specific to databases using subject headings and free-text words related to CWP, including fibromyalgia along with self-management interventions and qualitative methodologies (Appendix C, http://links.lww.com/PAIN/C119). There were no language restrictions.

### 2.3. Eligibility criteria

#### 2.3.1. Inclusion criteria

The SPIDER tool^13^ for qualitative evidence synthesis is used to outline the eligibility criteria.

##### 2.3.1.1. Sample

Adults (18 years of age or older) with CWP (including fibromyalgia) were included. Studies were included based on the authors' indication of including individuals with CWP or fibromyalgia. Chronic widespread pain is defined as persistent pain affecting multiple regions of the body, including both sides of the body, above and below the waist, and the axial skeleton.^56^ Fibromyalgia is characterised by CWP, often accompanied by cognitive disturbances, such as difficulties with concentration and attention.^12^

##### 2.3.1.2. Phenomenon of interest

Peoples' experiences of self-management interventions for CWP, including fibromyalgia. Self-management interventions are defined in the following way (adapted from Miles et al.^35^):

A self-management intervention

(1) has the broad goal of improving participants' health status or quality of life with scope for improvement in patients managing their own health;
(2) aims to increase participants' skills and knowledge and to enable participants to deploy these enhanced skills in aspects of their lives beyond the intervention;
(3) is directed at patients; and
(4) includes multicomponent interventions, eg, included exercise and a psychological component.

Following the definition of Miles et al.,^35^ to be considered self-management, the intervention must be composed of at least 2 components from the following 5: psychological (including behavioural or cognitive therapy, or an alternative approach that taught skills), mind–body therapies (MBT) (including components, such as relaxation, mediation, or guided imagery), physical activity (any form of exercise), lifestyle (such as dietary advice and sleep management), and medical education (such as information to support peoples' understanding of their condition and effective use of medication).

##### 2.3.1.3. Design

Primary qualitative studies or mixed-methods studies that include a qualitative component, provided the qualitative component was reported in enough details to inform the analysis.

##### 2.3.1.4. Evaluation

Descriptions of peoples' experiences of self-management interventions used for CWP and researchers' interpretations.

##### 2.3.1.5. Research type

Primary qualitative and mixed-methods research published in peer-reviewed journals. There was no language restriction.

#### 2.3.2. Exclusion criteria

Studies focusing on single component management interventions (eg, exercise or relaxation alone) and studies of interventions to support self-management of multiple coexisting conditions (eg, fibromyalgia and chronic abdominal pain) were excluded. Studies that contained only very limited qualitative data (eg, surveys with limited free-text options) were also excluded. Any decision to exclude an article on such grounds were discussed and agreed amongst the research team.

### 2.4. Study selection

Two searches were run, one in May 2022 and an updated one in November 2023. The screening process was performed independently [A.G., B.Y., X.Y.H.]. After the removal of duplicates, the reviewers screened titles and abstracts of identified articles based on the inclusion criteria. The full articles that matched our criteria were then obtained. Where the full article was proved to be a conference abstract, an attempt was made to locate a full published report of the study. If no full report was available, the study was excluded. Foreign language articles were translated into English using Google Translate. Any disagreements regarding inclusion that were unresolved were arbitrated by a third reviewer.

### 2.5. Data management

EndNote X9^53^ was used to manage references identified from the databases and for the removal of duplicates. Rayyan^42^ and Covidence^15^ were used to facilitate and coordinate the double-screening process. NVivo (Version 12)^40^ was used to manage the coding process.

### 2.6. Data extraction

A data extraction form was created and piloted for the characteristics of the included studies and qualitative data for synthesis. Study characteristics and qualitative data were extracted by one reviewer [B.Y. or X.Y.H.] and checked by a second reviewer [X.Y.H. or A.G.]. Any discrepancies identified were resolved by discussing with a third reviewer. Qualitative data were imported into NVivo for analysis.

### 2.7. Quality appraisal

A quality appraisal of the articles was carried out by 2 members of the review team [B.Y. or X.Y.H.] and checked by an alternative second reviewer [X.Y.H. or A.G.]. The Critical Appraisal Checklists (CASP)^45^ for qualitative studies was used to appraise the articles. One of the CASP items, “How valuable is the research,” was appraised based on how valuable the research was for our specific research questions, and the judgement was not made on how valuable the original studies were. Articles were not excluded on the basis of appraisal rating, rather consideration of appraisal rating was incorporated into the analysis and discussion.

### 2.8. Data synthesis

Thematic synthesis^54^ was used in this review because it is specifically created for designers or developers of interventions and policy makers.^5^ It provides clear stages in its method, and its explicit focus on open line-by-line coding ensures that descriptive and analytical themes can be transparently traced back to primary studies and source data.^54^ One researcher [B.Y.] coded the data line by line, then inductively identified preliminary concepts, grouping them into descriptive themes and subthemes. To develop analytic themes, the researcher [B.Y.] considered the relationships between the descriptive themes and explicitly focused on potential overarching concepts with explanatory power. For the 5 additional articles identified in an updated search, another researcher [X.Y.H.] inductively identified concepts and grouped them into themes, which were comfortably aligned with the analytical themes. Two researchers [X.Y.H., A.G.], who had also read all of the included articles, agreed the analytic themes and shared them for discussion with the whole team, including our Public Advisory Group (PAG) in research meetings. These larger discussions were used to ensure that the themes and subthemes were clear and held meaning for those in our team with lived experience. To ensure balance, a rigorous search for negative cases was performed as part of translating concepts between the studies.

### 2.9. Patient and public involvement

The idea to work towards accessible, effective, self-management support for CWP including fibromyalgia came directly from working with person's with lived experience (PWLEs) of CWP on a parallel project on low back pain. For this specific review project, we worked with a group of 4 public contributors with a range of experiences of CWP, including fibromyalgia, to form our PAG. They were actively involved in discussion of the review aim and design at the protocol development stage. They participated in multiple discussions of initial descriptive and analytical themes and, importantly, agreed the final analytic themes, the thematic model, and the overarching story the analysis tells. For example, public contributors felt strongly that people opting for group-based interventions have particular characteristics, and groups will not suit all. They emphasised the importance of age and ethnicity in framing people's experiences with CWP. Their feedback was also taken into account when we interpreted study limitations and drew conclusion.

## 3. Results

### 3.1. Identification and selection

**Figure 1.**
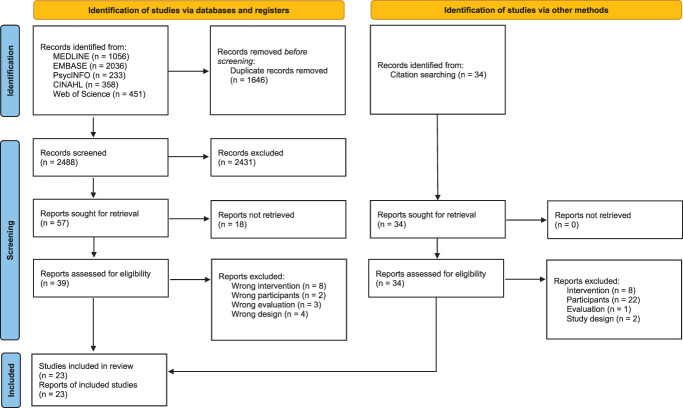
PRISMA flow diagram. PRISMA, Preferred Reporting Items for Systematic Reviews and Meta-Analyses.

A flowchart detailing the selection of studies is presented in Figure 1. From 4168 citations retrieved in the searches, 1646 duplicates were removed, leaving 114 for title and abstract screening. A total of 23 eligible studies were included involving 321 (ranging from 1 to 44) participants.^2,3,6,8,9,14,20,22,23,29,32,34,35,37,38,39,41,43,44,46,48,50,51^ Although one of the studies only included 1 participant, it extensively explored a 9-month self-management journey of a female subject with fibromyalgia in a case study, with sufficient data followed a comprehensive, interpretative, phenomenological analysis.^39^

### 3.2. Main study characteristics

Characteristics of the included studies are presented in Table 1. Most studies were conducted by teams from the United Kingdom^6,33,39,43,44,50^ and Sweden.^9,19,21,22,31^ Apart from 3 studies that recruited participants with CWP^9,19,21^ and one defined as multisite MSK pain,^37^ all others interviewed people with fibromyalgia specifically,^2,3,6,8,14,23,29,32,34,35,37,39,41,43,44,46,48,50,51^ age from 20^51^ to 83 years,^44^ with most of them being female (238 of 260, 92%), including more than half of the studies (12 of 23) on purely female participants.^2,3,22,23,29,32,39,41,43,46,48,51^ Only 2 studies reported ethnicity of included participants.^33,41^ Although no restriction was put on delivery of intervention, all but 2 studies (91%) delivered the interventions as a group based.^2,3,8,9,14,22,23,29,32,34,35,37,38,39,41,43,44,46,48,50,51^

**Table 1:** Characteristics of the included studies.

Country	Study	Study design	Setting	Participants: size, diagnosis, age range (mean, SD, y), sex	Intervention				Analysis method
					Group-based or not	Name: Techniques^$^	Duration, timing	Intervention providers (eg, number, profession, training)	
UK	Bee 2016	MM—QnRCT	Primary care	44 fibro, NR (58, 13), 33F/11M	N	N/A: Prescribed exercise and/or cognitive behavioural therapy	6 m, at least twice/wk, 7 weekly sessions, 3 m/6 m post assessment	Personal trainers, experienced and accredited therapists	Framework
	Nizza 2018	Qual—CS	Community service	1 fibro, 47, 1F/0M	Y	N/A: Develop self-management skills, such as relaxation, mindfulness, goal-setting, pacing, and sleep hygiene	4 w, NR	NR	Interview: IPA, drawings: visual analysis method
	Pearson 2020	Qual	NHS Foundation Trust	9 fibro, NR, 9F/0M	Y	FSMP*: Condition-specific, patient-centred education (increasing knowledge and understanding of the condition, medication, goal setting, pacing, dietary advice, sleep hygiene, relaxation) and exercise advice	4/6 w, weekly	PT, OT, specialist nurses, and dieticians	Framework
	Pearson 2022	MM—QnfRCT	City and rural community GP/hospital	13 fibro, 23–83 (48.15, 16.69), 11F/2M			6 w, weekly	PT, OT	Qualitative description
	Sharma 2022	MM—Qual service evaluation with observational study	RLHIM, part of UCLH NHS Trust	14 fibro, NR, NR		Multicomponent fibromyalgia service: pain management, OT, CBT. Patients can also be referred for PT, acupuncture, and/or dietetic input if required	9 mo, one introductory session, followed by 9 sessions, with 3 FUs at approximately 3, 6, and 9 mo	NR	Inductive thematic analysis under constructivist epistemological approach
	Mcllroy 2022	Qual—service evaluation	One NHS Trust	6 HCP, 4F/2M; 7 fibro, or widespread pain and fatigue (32-56), 7F/0M	Y	FAME: outpatient programme to improve function and QoL by facilitating self-management and physical activity	12 w, weekly	PT, pain nurse, psychologist, dietician	Inductive thematic analysis
Sweden	Bremander 2009	Qual—GT	Secondary care	16 CWP, 29-64 (46, NR), 13F/3M	Y	N/A: Multimodal treatment with a cognitive approach and a biopsychosocial perspective on pain, body awareness therapy, pool exercise, qigong, and individual counselling	6 m, 3 w/3 m/6 m	Physician, psychologist, OT, physical therapist	Reformulated grounded theory
	Feldthusen 2022	Qual	5 primary care centres	19 CWP, 26-60 (median 47), 16F/3M		N/A: Cocreation of a health plan for sustainable physical activity	12 m, 2 meetings with an interval of 2 w	PT	Content analysis
	Goksor 2022	Qual	Primary care	12 CWP, 25-72 (NR), 12F/0M	Y	Pain school: lectures, group discussions, relaxation exercises, and an introduction to physical activity and exercise	14 w, 4 weekly	PT, OT	Content analysis
	Gustafsson 2004	Qual	Referred by social security office	16 fibro, 23-59 (43, NR), 16F/0M	Y	N/A: education, group discussion, physical training, and individual guidance^†^	NR, NR	PT, social workers	Grounded theory using constant comparison
	Mannerkorpi 2003	MM—QnRCT	University hospital	19 fibro, 28-59 (45, NR), 19F/0M	Y	N/A: physiotherapy group treatment comprising pool exercise and education	6 m, pool exercise weekly and educational programme on 6 occasions	NR	Phenomenological life-world approach
Norway	Mengshoel 2021	Qual—PAR	Hospital	20 fibro, NR, unclear	Y	PROP: group discussions, individual exercises, and a written diary	NR, 8 full-day seminars arranged in intervals of 2-4 w	Physician, PT psychologist, OT, nutritionist, social worker	Thematic analysis
	Singstad 2020	MM—QnRCT	A municipal healthy life centre	6 fibro, 20-50, 6F/0M	Y	VTP: mindfulness training, values-based action, and various creative methods	4 m, 10 sessions	VTP course leaders	Malterud's approach to systematic text condensation
	Misje 2023	Qual—phenomenology	A multidisciplinary outpatient clinic	8 multisite MSK pain, 29-56, 7F/1M	Y	MB programme: rehab, body awareness, relaxation, and grounding exercises; cognitive techniques; Tibetan yoga; mindful meditation	3.5 h over 4 w	2 CBT nurses, 2 PTs experienced in body awareness approaches	Systematic text condensation
Canada	Bourgault 2015	MM—Qual nested in a RCT	2 university-affiliated settings	16 fibro, NR, NR	Y	PASSAGE program: exercise therapy and educational/psychological tools for self-management	The first 8 sessions over 11 w, 9th session 6 m later	HCP (attended a training session, provided with a course manual)	Thematic analysis
	Lagueux 2021	MM—Obs and qual	Occupational therapy settings in 2 pain management clinic	6 fibro, NR, 6F/0M	Y	FCLR: perform their own occupational analysis (daily activity patterns, physical and mental well-being	13 w, 6 group based and 3 individual OT sessions	OT	Thematic content analysis
Brazil	Costa Guedes Miranda 2016	Qual—participatory approach	Community	11 fibro, NR, NR	Y	Integrated community therapy	Over 9 m with 3 × 3-month phases: 1st stage (adaption): health education activities weekly; 2nd and 3rd (transition) stages: weekly	NR	Content analysis
	Oliveira 2019	Qual	State University	12 fibro, 33-73, 12F/0M	Y	N/A: health education, exercises, nutritional orientation, therapy		Interdisciplinary group, psychologists, under the coordination of the psychology department	
Spain	Arfuch 2021	Qual with a linked RCT	11 primary care centres	19 fibro, 46-79 (61.8, 8.4), 19F/0M	Y	N/A: health education; physical exercise; CBT	12 w, weekly	GP, PT, psychologist, head nurse	Thematic analysis
	Arfuch 2022	Qual	5 primary care centres	10 fibro, 58.5 (45, 73), 10F/0M	N	N/A: health education; physical exercise; CBT	12 w, weekly	Female GP, PT, psychologist, head nurse	Thematic analysis
Denmark	Rasmussen 2017	MM—Qual nested in a RCT	Outpatient clinic	17 fibro, NR (42.8, 12.9), 17F/0M	Y	N/A: lectures, group discussions, and training sessions	2 w, daily for 10 consecutive weekdays	Rheumatologist, pain psychologist, nurses, PT, OT, DFA representatives	Constant comparative, applying open, axial, and selective coding
Finland	Sallinen 2021	Qual	Rheumatism Foundation Hospital	20 fibro, 34-65, 20F/0M	Y	N/A: lectures, group discussions, physiotherapy group exercises, and individual treatments	17–20 d, NR	NR	Stepwise applying the ideas of Polkinghorne, Labov, Waletsky, and Reissmann
Northern Ireland	Courel-Ibáñez 2023	MM—Qual nested in a feasibility proof-of-concept trial	Patient support group setting	NR (probably 5 fibro), NR, NR	Y	N/A: rehabilitation programme followed by physical activity	Weekly 1-1.5 h session, 4 wk	A PT and a sports scientist	Reflexive thematic analysis

* Where there are multiple studies of the same intervention, cells were merged cross rows. $ Characteristics of the multicomponent self-management interventions are provided in Appendix D, http://links.lww.com/PAIN/C119.

†A vocational rehabilitation programme that patients were referred by social security office.

C, cognitive; CBT, cognitive behavioural therapy; CS, case study; D, day; Di, digital; DFA, Danish Fibromyalgia Association; F, female; FCLR, French-Canadian Lifestyle Redesign; fibro, fibromyalgia; fRCT, feasibility RCT; FSMP, Fibromyalgia Self-Management Programme; FU, follow-up; GT, grounded theory; HCP, healthcare professionals; IPA, interpretative phenomenological analysis; M, male; m, month; MB, mind and body; MM, mixed methods; NR, not reported; OT, occupational therapy; PAR, participatory action research; Phy, physical activity; PROP, patient-centred, recovery-oriented programme; Psy, psychological; PT, Physiotherapist; QnfRCT, qualitative study nested in a feasibility RCT; QnRCT, qualitative study nested in a RCT; QoL, quality of life; Qual, qualitative; RCT, randomised controlled trial; VTP, vitality training programme; w, week.

There was in general a lack of reporting of detailed research methods. Many studies did not report their inclusion or exclusion criteria^9,22,31,34,39,43^ or only provided relevant information in separate trial articles.^6,19,44,51^ Methods of recruitment were mainly through providing written/oral information^3,6,19,21,43,51^ or through advertisements^39^ or existing groups.^33,37^ Apart from 9 studies that reported using purposive sampling^2,3,6,35,38,43,44^ or open sampling by inviting all that completed the treatment,^31,46^ the rest did not provide a clear sampling method. Data collection was mainly through semistructured interviews^3,6,9,19,21,22,28,34,37,38,39,41,43,44,51^ or with some forms of observation.^36,43^ Others were through focus group,^2,8,34,46,50^ phenomenological interviews,^31^ or narrative life story interviews.^34^

Most intervention programmes/packages provided sufficient information on techniques of the interventions. The majority of interventions explored covered at least 3 components from the following 5 elements; psychological, mind–body therapies, physical activity, lifestyle, and medical education. Only 1 study included just 2 elements (Appendix D, http://links.lww.com/PAIN/C119).

### 3.3. Methodological quality assessment

Table 2 provides a summary of the critical appraisal of the methodological quality of the included studies. Overall, 18 studies were globally rated as having high quality and 3 of moderate quality. Only 5 studies fulfilled all 10 criteria of the CASP checklist.^3,6,9,38,46^ Insufficient documentation and consideration were given to the Q6 “relationship between researchers and participants” in many (12 of 23) of the studies. A global rating was given by the research team to show the overall quality. The studies were rated as of low, moderate, or high quality, based on a detailed assessment of Q4, Q5, Q7, and Q8.^38^

**Table 2:** Critical appraisal of the methodological quality of the included studies.

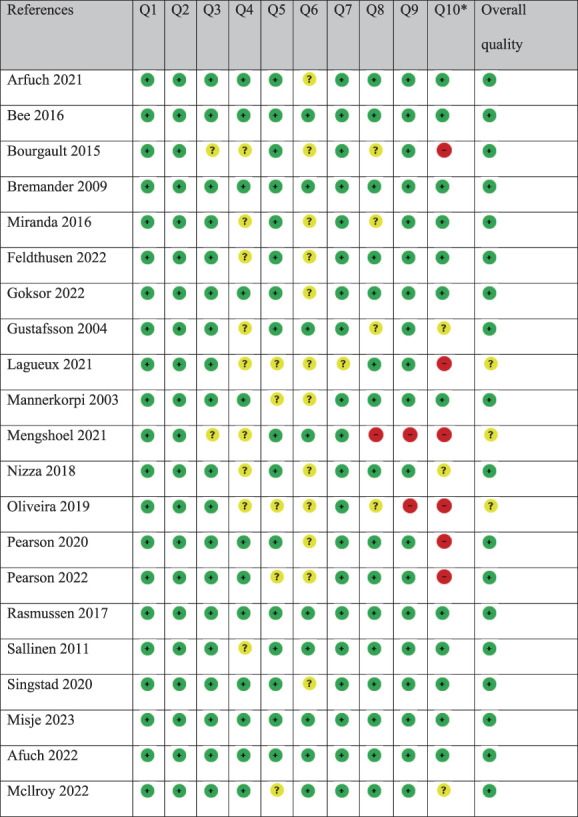
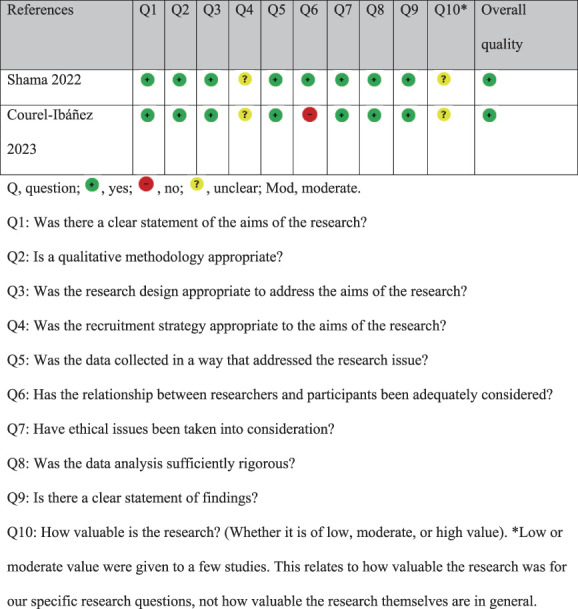

### 3.4. Synthesis

**Figure 2.**
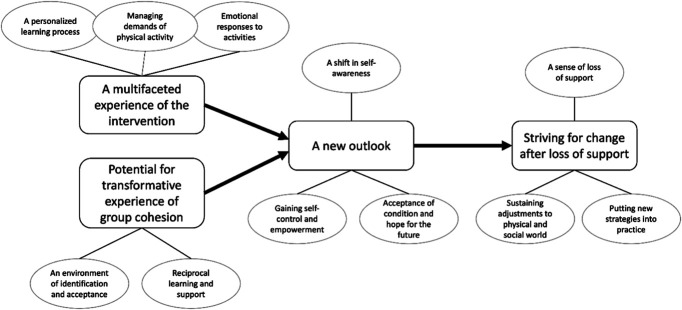
A model of analytic themes and subthemes.

Four overarching analytic themes were developed: a multifaceted experience of the intervention, the potential for transformative experience of group cohesion, a new outlook, and striving for change after the loss of support. The respective subthemes are described in the following sections. A model representing the themes is presented in Figure 2.

#### 3.4.1. Theme 1: a multifaceted experience of the intervention

By their nature, self-management interventions comprise a range of differing components and strategies. This variety was reflected in the diversity of what appeared central to people's experiences of intervention activities. Key findings, to be explored further below, were that perceptions of personalisation and fit appeared to be important to participants, there was a tension around the demands and benefits of exercise, and the course content had a range of emotional impacts. These 3 areas appeared linked by a subtle, underlying notion of alignment: alignment through personalisation, tensions when exercises felt misaligned with capacity, and key aspect of positive emotional experience being driven by validation, through the aligning of professionals and others with the individual and their experience of pain. The positive aspects of these experiences across the areas, appeared to be the beginning of a process of change experienced by some participants that facilitated subsequent changes in thinking and behaviour.

*A personalised learning process*^2,6,8,9,20,22,29,31,34,36,39,41,44,46,48,51^: Participants often perceived the intervention activities as personalised, flexible, and delivered by credible and attentive professionals. Participants' positive perceptions of personalisation appeared to be driven by activities that aligned with their understandings about their condition, how effective they were finding the intervention, and goals, whether familiar or novel. Personalisation was also linked to experiences of the professionals that delivered the activities, such as their perceived credibility and accessibility, and their understanding, knowledge, and authority. In addition, the relationship they formed with professionals contributed to perceptions of personalisation, for example, the extent to which professionals were available, interested, and willing to listen. Conversely, perceptions of a lack of engagement from, or credibility of, professionals adversely impacted reported feelings of personalisation.

I thought it was really good. It worked great. Because … Then I knew all the time, that I had a plan and it was just for me. I didn't need to compare myself with anyone else, and so on. It was aimed at me. (Participant received individualised self-management)^19^

The co-creation of the health plan for physical activity was described as the most valuable part of the intervention. The respondents experienced this as a new model that they had not previously encountered within healthcare. (Author)^19^

Where some felt less relevance, this seemed related to a lack of “fit” between participants' models of their condition and particular models used in treatment approaches.

Lack of relevance was framed in different ways, but included at its core two key factors: a lack of fit with participants' entrenched illness perceptions and a lack of fit with the self. Participants' narratives revealed a lack of knowledge regarding the goals and remit of CBT and thus an initial lack of understanding regarding its “fit” with a health condition predominantly attributed to physical causes. (Author)^6^

*Demands of physical activity*^2,9,22,44^: The physical component within the self-management interventions was unrealistic in some participants' circumstances. These participants described the physical activity suggestions as potentially too much, presenting a risk of exacerbating symptoms. The authors of the studies often summarised this difficulty, highlighting the complexity of the need to manage perception of risk and difficulty in finding the right level of physical activity.

The problem was that they insisted too much on “walk, walk, walk.” But what if I do not like so much this type of work out and I prefer something else? I think they do not really understand our limits […]. I think they did not adjust their methods to our condition. However, it was not deliberately, I believe […]. (Participant).^2^

The informants found it difficult to find a suitable level of exercise due to pain and either exercised too much which increased their pain or they took no exercise at all and treated the pain exclusively by means of rest. (Author).^9^

*Emotional responses to activities*^2,3,6,8,9,20,22,23,32,35,37,39,41,44,46^: Participating in intervention activities generated emotional responses that, for some, were significant in the overall process of supporting self-management. Participants often reported experiences of relief and relaxation, both physically and psychologically, as they shared their untold illness experiences and felt believed, fostering trust and security. Some participants spoke about the intervention as a respite in their lives and a means to shed emotional burdens. These participants described the intervention as “a breathing space in life” and as “[getting] rid of things we carry inside the backpack.” Trust and security could be further bolstered by access to knowledgeable healthcare professionals. Feelings of success, accomplishment, and well-being resulted for those who were fulfilling activity plans. Overall, these positive emotional responses, along with the contrast with past experiences, appeared to be important in allowing participants to gain a new outlook and facilitated a transformative shift in self-awareness.

I wanted to say that, for me, this whole experience has been incredibly positive, especially emotionally. (Participant)^2^

It appeared that, during relaxation, many subjects were able temporarily to leave their world of pain and stress and feel peace of mind. The significance of relaxation was understood against the background of the respondents' descriptions of their common experience of a painful, tense body. (Author)^31^

Reflecting over their health together with a knowledgeable person who acknowledged their limitations and circumstances brought an experience of trust and security. (Author)^19^

In some studies,^2,3,6,14,20,44,46^ a few participants reported negative emotional responses to intervention activities. For some, this was frustration at not being able to change in desired ways. For others, negative emotional responses were driven by specific elements of course content, particularly material on acceptance, or intervention with limited time input, which led some to feel upset and angry.

Others were frustrated by their own inability to move on and change their life plan. (Author)^9^

Although the acceptance and grief cycle were perceived as helpful, some participants found discussing this overwhelming and others described feeling upset or angry. (Author)^44^

#### 3.4.2. Theme 2: potential for transformative experience of group cohesion

The second theme highlights the potential impact of group-related intervention activities, when perceived as positive they can provide transformative experiences through interpersonal interactions. All but 2 studies delivered the interventions as a group based.^2,3,6,8,9,14,22,23,29,32,34,35,37,38,39,41,43,44,46,48,50,51^ Participants often found acceptance, respect, and support from peers, sharing and learning from each other's experiences. This sense of cohesion and belonging appeared to be key in facilitating a new outlook on managing their condition, contrasting with past feelings of isolation and misunderstanding.

*An environment of identification and acceptance*^2,3,8,9,22,29,32,34,37,39,41,46,48,51^: This subtheme emphasises the cultural and environmental factors in the intervention that appeared to work to foster group cohesion and personal transformation. Acceptance and respect from peers, along with a sense of identification, were defining features. Participants often felt welcomed, valued, and connected, sharing common problems and feelings. This safe and open environment facilitated honest discussions, contrasting with past experiences, and appeared pivotal in individuals' self-management journey. The climate of acceptance and the bond formed within the group played a crucial role in facilitating positive changes in their condition's self-management.

…  because we had such a great time together … and you could sort of say what you felt and such … and everybody somehow accepted everybody else. (Participant)^9^

The community therapy circles afforded an opportunity to speak, offering people a welcoming environment, of active listening and respect for each other. Thus, people felt comfortable to discuss their life situations. (Author)^36^

You don’t feel judged …. I was putting on this poker face, so coming here, I didn't have to put on that face, I could be honest and say I'm in pain or how I felt. (Participant)^33^

*Reciprocal learning and support*^2,3,8,9,22,29,32,34,35,37,39,41,46,48,51^: The reciprocal understanding, support, and learning within intervention groups helped many participants interpreting their experiences and adopting new self-management strategies. This subtheme goes beyond acceptance, respect, and identification to explain how participants often adopted a mutual understanding to actively support each other and share insights during group interactions. Support received was motivating, and sharing their experiences to help others was meaningful. However, some participants struggled to accept support, distancing themselves from peer experiences, or feeling overwhelmed by others' stories.

You know a lot about each other and can discuss many things and have similar experiences … and if one person has experienced something that the other person has not, that person can share her or his experience. (Participant)^9^

Sharing experiences and creating new meanings and patterns appeared to have two parallel directions, an outward and an inward, one social and the other personal. While listening to others' experiences, the respondents appeared to have directed a part of their consciousness toward their own life-world. It appeared that many incentives for new patterns of thinking and acting were discovered during this process. (Author)^31^

Discussing with people who are going through the same thing, make you feel not so weird and shamed. Because this disease is incomprehensible. The program has helped me understand that I am not the only person who is going through it. (Participant)^3^

Although the group experience was reported positively by most, in some studies,^2,3,31,34^ individuals expressed negative feelings when there was inconsistent attendance or felt that the group intervention could not be tailored to individuals. Some described the group approach as emotionally burdensome, leading them to feel “overwhelmed” by listening to others' problems or prompting them to distance themselves from individuals whom they perceived to have a “passive attitude towards life,” or they found to be a barrier to personal change.

In the groups, for example, I always take the role of the funny guy. It is my shield. I am not that expressive …. There were too many people in the group; too many problems to share; too many mouths to talk; too many thoughts to be said. It is complicated. (Participant)^3^

Some patient participants reported that their peer support network was threatened by inconsistent attendance of some attendees and that this affected their sense of a shared community… Patient participants reported that some staff were not able to tailor information in the group setting. This affected their attitude towards and perceived effectiveness of FAME. (Author)^33^

#### 3.4.3. Theme 3: a new outlook

This theme highlights how participants often developed a new outlook, encompassing transformed perspectives of themselves, their condition, and their lives. For these individuals, these important changes in thinking following intervention experiences appeared to be an important aspect of the impact of interventions on improved self-management.

*A shift in self-awareness*^2,3,6,8,9,20,22,23,32,35,37,38,39,41,46,51^: Many participants experienced transformative changes in self-perception and self-imposed demands. They adjusted their expectations, prioritized self-care over meeting others' expectations, and embraced self-compassion. This shift towards self-focus, contrasting with past tendencies to neglect personal needs, allowed them to act differently and reduce negative feelings about falling short of expectations. In addition, participants often experienced improved self-esteem, self-image, and self-insight, accepting themselves physically and perceiving increased strength and personal growth. This increased confidence in self-management skills and self-care abilities replaced feelings of inadequacy, enabling the setting of boundaries, developing strategies, and enhancing interpersonal relationships.

I think I'm improving with myself, I'm looking at myself more closely in the mirror, viewing myself differently. (Participant)^36^

Several said that the course and the period afterward increased their respect for and acceptance of their own needs, and they gave themselves permission to attend to their needs and take themselves more into account. Many described how they had downplayed their own needs in the past, and admitted that this did not work well. (A)^51^

*Gaining self-control and empowerment*^2,3,8,32,34,37,39,41,46,48,51^: For participants who attained enhanced control over thoughts, actions, and symptoms, this often resulted in a feeling of empowerment and agency. They experienced increased self-control, autonomy, and responsibility, taking an active role in managing their condition and believing in their ability to effect positive changes.

It's good to know that the disease doesn’t get to decide everything, but that I can actually decide some things as well. And make decisions that affect the disease, not only that the disease affects me. This means that I myself have some control. (Participant)^51^

What changed and was important to Jane was that she felt empowered by the pain management programme to challenge her existing relationships and make practical changes to her life, enabling her to regain a sense of mastery. (Author)^39^

*Acceptance of condition and hope for the future*^2,6,8,9,20,22,23,29,31,34,37,39,41,43,46,48,51^: This subtheme describes a new outlook many participants gained on their condition and their future. It encompasses a new understanding of pain, including greater awareness of activity's impact on health and body signals related to pain, as well as insights into psychological well-being. Participants new outlook often involved acceptance of their condition; this acceptance appeared to support the adaptation of strategies to live with pain. Some held hope for symptom improvement and a positive future. In addition, it was common for participants outlooks on life events, social roles, and identities to undergo positive changes as part of the intervention process.

I've learned to recognise signals that help me not to push myself too hard. That’s perhaps the most important thing. Noticing my stress level when it comes. I didn’t do that before. It doesn’t help for others to say it, you need to recognise it yourself. I think the course was very good at this—learning to notice it yourself. (Participant)^51^

Participants who had denied or challenged pain recounted how they had gradually begun to re-engage with their condition and legitimise their symptoms. (Author)^6^

I have to acknowledge my duties in a fast-paced work environment. The way I deal with stress, the way I work and think about stress and what strategies I have to manage stress all contribute to improve my work situation. (Participant)^37^

However, hope and acceptance were not universal. In a smaller number of studies,^21,48^ authors described how some participants remained hopeless, or working with others who were more severe, led to concerns and anxiety about the future.

Some participants never felt free of pain and did not think that their pain would ever change. (Author)^21^

However, seeing others with more severe functional disabilities or depression also raised some contradictory thoughts; anxiety related to the future, occasional hopelessness and despair and fear of mental health problems. (Author)^48^

#### 3.4.4. Theme 4: striving for change after the loss of support

The final theme centres on the postintervention phase, where participants coped without formal healthcare support, tried to implement learned self-management skills and faced barriers to self-management in their day-to-day lives. It focuses on factors influencing the sustainability of changes in self-management after the intervention.

*A sense of loss of support*^2,9,22,43,46^: Participants often faced challenges when interventions ended, experiencing feelings of abandonment and longing for the support they had during the programme. They expressed a need for continued reinforcement of the intervention's information. The absence of group interactions with fellow participants was particularly missed. Many participants believed that interventions should be extended and supported further, as the postintervention phase remained a crucial period of ongoing change requiring continued assistance.

I feel more and more when I am alone that you get help to start a process but not to continue it and you end up in the middle of somewhere and then it abruptly ends. (Participant)^9^

Participants had been missing the group fellowship after the course, and expressed a wish for further follow-up so that they could meet again to keep in touch and also reinforce the contents of the course together. (Author)^46^

*Putting new strategies into practice*^2,21,23,31,34,36,39,41,43,46,51^: Participants described implementation of learned self-management strategies, documenting both successes and failures. They engaged in new physical activities, incorporated relaxation skills into their daily routine, and sought alternative ways to manage their condition. The theme emphasizes the differences between these new strategies and their past approaches to self-management.

I am in pain from the moment I get up. I know that the pain is not going to disappear, but I try to avoid it somehow. So, I keep taking my medication, and I try to take short breaks and rest if I feel tired. I also practice physical activity regularly. I started swimming, and I go to the sauna and the jacuzzi. Honestly, I am doing very well now. I try to do it every day for at least one hour. (Participant)^2^

Participants were striving to incorporate new good habits from what they had learned during rehabilitation, which included structured planning of their everyday life according to their daily condition to save energy for the most important things. They were striving to focus on the positive and prioritise to do what gave the most joy. (Author)^46^

*Sustaining adjustments to physical and social world*^2,6,8,9,20,22,23,31,34,36,39,41,46,51^: This subtheme describes participants' readjustment to daily life and the barriers and facilitators they encountered in implementing and maintaining self-management changes. Psychological, social, and environmental factors played a crucial role in this process. Participants' reorientation was influenced by new knowledge and skills gained from the intervention. Successful changes often required overcoming old habits, adapting routines, and dealing with limited resources. Improved relationships, social support, and the significance of participants' social identity were highlighted. Psychological factors like knowledge, memory, motivation, and confidence also influenced sustainability, while pain symptoms and health issues posed barriers.

You do not only work with this, of course you need to work with them too but … there is also everyday life and your children, you do not have as much time as you would like to think about yourself. And at the hospital, you had that week to rest and work with yourself. There was nothing to disturb you. (Participant)^9^

Limitations for physical activity in the form of limited access to warm water pools (introduced in the programme), or financial constraints preventing participation in organised training programmes, were additional obstacles to becoming more physically active. (Author)^46^

A lot has changed for the better at home, because I understand it's not their fault and they don't know what I'm feeling. (Participant)^36^

## 4. Discussion

This systematic review provides unique insights into the processes by which aspects self-management interventions are most helpful, or not, when offered to people with CWP, including fibromyalgia. For some participants, the multifaceted nature of these self-management interventions provided a personalised and credible experience, although physical components posed challenges for some, indicating a need for greater customisation. The emotional responses to intervention activities appeared to facilitate subsequent changes in behaviour and thinking. Group-related processes were often important, where acceptance and mutual support among participants seemed to foster a new outlook on condition management, contrasting with prior feelings of isolation. Reciprocal learning and support within the groups helped participants interpret their experiences and develop new self-management strategies. Some experienced a transformative shift in their outlook, gaining self-control and a sense of empowerment. This led to an increased acceptance of their condition and hope for the future, although not universally. The end of the intervention often posed challenges, with participants feeling a sense of loss and struggling to sustain learned strategies.

The self-management interventions reviewed provided the context and resources for a highly complex, multifaceted process, with different aspects likely to affect different individuals in different ways. It seemed that for those who found benefit, there was a somewhat emergent effect of the components working together to create what we termed a “new outlook.” However, it was also clear that certain aspects caused problems, possibly blocking this process for some. For instance, although exercise/physical activity has been shown to be effective in fibromyalgia,^24^ it was one of the areas that was more challenging for some participants. This is consistent with other studies that have found that adherence to exercise is difficult due to increased CWP/fibromyalgia symptoms.^11^ There is a tension for intervention developers: how to include techniques with known effectiveness such as exercise and deliver them in a way that enables access to their potential benefit, for as many as possible.

Looking across our analytic themes, the impact of the group setting was also complex. It was clear that some found the groups setting to be important in a number of ways, a finding consistent with 2 previous qualitative systematic reviews on self-management for chronic pain.^16,52^ However, the loss of support of the group at the end of the interventions appeared to have a strong negative impact for some. Providing, then removing, support should be considered carefully. Ideally, tailored, long-term, adequate, and cost-efficient ways of maintaining aspects of positive support could be built in as part of the delivery of the interventions.^17^ There is evidence that peer-led support groups following formal programmes can be helpful.^18^ A network of peer social support may bring beneficial impact on individuals with fibromyalgia, improving their physical, mental, and social welfare and enabling them to more effectively cope with their condition.^47^ Technology may also facilitate this process (eg, using text message groups, people can self-maintain after the courses end). Relatedly, there were other struggles reported with implementing new approaches beyond the course (eg, overcoming old habits, dealing with limited resources). Problems with the lack of support following pain management programmes has been reported elsewhere,^18^ and Spink et al., in their review of qualitative studies of self-management for chronic musculoskeletal pain, concluded that ongoing support is often required.^52^ In a similar systematic review of qualitative studies on self-management, in this case including all forms of chronic pain, Devan et al.,^16^ also conclude that sustaining motivation is a key barrier to self-management. Addressing these sustainability issues regarding postcourse integration may require an overt focus on what will happen at the end of the course in the material from the outset.

### 4.1. Strength and limitations

The quality of the included studies varied but most were high or moderate. To improve robustness and reliability of the analysis and our interpretation, we had a clear coding and analysis procedures, involved other researchers and public contributors in developing and regrouping themes and interpretation of the findings, and resolved discrepancies through consensus meetings. None of the included studies reported looking specifically for negative reports; however, we searched for disconfirming quotes or discussions.

Limitation of the included studies: Some studies were published 20 years ago, which may not fully reflect the contemporary context and peoples' experiences. Most of the included studies were conducted in European countries and seldom reported ethnicity or socioeconomic status. They seldom clarified whether the interviews were conducted in English or described the translation process. Most of the participants from the original studies were female, which restricted the generalisability of our findings to male population. Although we used CWP, including fibromyalgia as our primary definition, the majority of participants in the studies had fibromyalgia, and only 4 studies used a broader chronic widespread/multisite pain definition. Thus, our findings apply primarily to the more severe fibromyalgia end of the CWP spectrum. The durations of all interventions and data collections reviewed are relatively short term considering the chronic nature of the condition.

In addition, of the studies included, more than 90% of the self-management interventions were delivered in groups. Our public coauthors with lived experience felt strongly that although our themes characterised the experience of those included in group-based interventions, there will be many for whom the group format would lead to exclusion. This should be considered when generalising our findings. Limited data on deprivation and ethnicity further constrain the ability to draw comprehensive conclusions. Studies employed different theoretical frameworks and data collection methods, which may present challenges in coherently synthesising the findings. Those who consented to be interviewed might have had more positive experiences with self-management. This may introduce a bias in favour of the interventions they received. In addition, there may be bias towards publishing studies with noteworthy findings, possibly leading to an underrepresentation of negative outcomes. Limitation of the review: We have used one particular published definition of self-management with a particular focus on multicomponent interventions.^35^ Although we felt that it is an important and useful definition which also kept the review to a manageable size, a broader definition could have led to the inclusion of more studies.

### 4.2. Implication for future research

Although the majority people with CWP reported positive experiences, our review has indicated certain factors that may limit the impact of self-management interventions for CWP, for instance, problems with physical exercises and struggles with acceptance. To ensure that self-management interventions have the greatest chance of benefiting those with CWP, it may be useful to explore adaptive interventions, where adjustments could be made rapidly for individuals, based on their progress and particular issues. For example, options to shift to online delivery for those who were struggling in the group setting may prevent dropout. Future research is warranted to explore diverse delivery formats to ensure inclusivity and effectiveness across various demographic groups, considering tailoring interventions to suit those who may struggle with physical activities or with groups.

## 5. Conclusion

In self-management interventions for CWP, personalised learning experiences and emotional responses appeared to drive behavioural and cognitive changes in people participating in the programmes. Group activities promoted acceptance and support, fostering new perspectives and improved self-management. Postintervention challenges emerged due to the lack of ongoing support. Although many had positive experiences, some benefited less, necessitating further research to identify and provide support for individuals for whom key aspects of these programmes have less impact.

## Conflict of interest statement

The authors have no conflicts of interest to declare.

## Appendix A. Supplemental digital content

Supplemental digital content associated with this article can be found online at http://links.lww.com/PAIN/C119.
