# Supplementary material for: Self-management interventions for chronic widespread pain including fibromyalgia: a systematic review and qualitative evidence synthesis
Source: Pain. 2024 Sep 17;166(3):e36–50. doi: 10.1097/j.pain.0000000000003379 (PMC11808693; doi:10.1097/j.pain.0000000000003379)
Supplement: Supplementary file 1 [file jop-166-e36-s001.pdf]

Appendix A. Preferred Reporting Items for Systematic Reviews and Meta-Analyses statement (PRISMA) 2020 checklist

Section and Topic	Item #	Checklist item	Location where item is reported
**TITLE**			
Title	1	Identify the report as a systematic review.	1
**ABSTRACT**			
Abstract	2	See the PRISMA 2020 for Abstracts checklist.	2
**INTRODUCTION**			
Rationale	3	Describe the rationale for the review in the context of existing knowledge.	3
Objectives	4	Provide an explicit statement of the objective(s) or question(s) the review addresses.	3
**METHODS**			
Eligibility criteria	5	Specify the inclusion and exclusion criteria for the review and how studies were grouped for the syntheses.	4
Information sources	6	Specify all databases, registers, websites, organisations, reference lists and other sources searched or consulted to identify studies. Specify the date when each source was last searched or consulted.	4
Search strategy	7	Present the full search strategies for all databases, registers and websites, including any filters and limits used.	4, Appendix C
Selection process	8	Specify the methods used to decide whether a study met the inclusion criteria of the review, including how many reviewers screened each record and each report retrieved, whether they worked independently, and if applicable, details of automation tools used in the process.	5
Data collection process	9	Specify the methods used to collect data from reports, including how many reviewers collected data from each report, whether they worked independently, any processes for obtaining or confirming data from study investigators, and if applicable, details of automation tools used in the process.	5-6
Data items	10a	List and define all outcomes for which data were sought. Specify whether all results that were compatible with each outcome domain in each study were sought (e.g. for all measures, time points, analyses), and if not, the methods used to decide which results to collect.	4
	10b	List and define all other variables for which data were sought (e.g. participant and intervention characteristics, funding sources). Describe any assumptions made about any missing or unclear information.	4
Study risk of bias assessment	11	Specify the methods used to assess risk of bias in the included studies, including details of the tool(s) used, how many reviewers assessed each study and whether they worked independently, and if applicable, details of automation tools used in the process.	6
Effect measures	12	Specify for each outcome the effect measure(s) (e.g. risk ratio, mean difference) used in the synthesis or presentation of results.	6
Synthesis methods	13a	Describe the processes used to decide which studies were eligible for each synthesis (e.g. tabulating the study intervention characteristics and comparing against the planned groups for each synthesis (item #5)).	6
	13b	Describe any methods required to prepare the data for presentation or synthesis, such as handling of missing summary statistics, or data conversions.	6

Section and Topic	Item #	Checklist item	Location where item is reported
	13c	Describe any methods used to tabulate or visually display results of individual studies and syntheses.	6
	13d	Describe any methods used to synthesize results and provide a rationale for the choice(s). If meta-analysis was performed, describe the model(s), method(s) to identify the presence and extent of statistical heterogeneity, and software package(s) used.	6
	13e	Describe any methods used to explore possible causes of heterogeneity among study results (e.g. subgroup analysis, meta-regression).	6
	13f	Describe any sensitivity analyses conducted to assess robustness of the synthesized results.	6
Reporting bias assessment	14	Describe any methods used to assess risk of bias due to missing results in a synthesis (arising from reporting biases).	6
Certainty assessment	15	Describe any methods used to assess certainty (or confidence) in the body of evidence for an outcome.	6
**RESULTS**			
Study selection	16a	Describe the results of the search and selection process, from the number of records identified in the search to the number of studies included in the review, ideally using a flow diagram.	7
	16b	Cite studies that might appear to meet the inclusion criteria, but which were excluded, and explain why they were excluded.	7
Study characteristics	17	Cite each included study and present its characteristics.	7-8
Risk of bias in studies	18	Present assessments of risk of bias for each included study.	8
Results of individual studies	19	For all outcomes, present, for each study: (a) summary statistics for each group (where appropriate) and (b) an effect estimate and its precision (e.g. confidence/credible interval), ideally using structured tables or plots.	8-15
Results of syntheses	20a	For each synthesis, briefly summarise the characteristics and risk of bias among contributing studies.	8-15
	20b	Present results of all statistical syntheses conducted. If meta-analysis was done, present for each the summary estimate and its precision (e.g. confidence/credible interval) and measures of statistical heterogeneity. If comparing groups, describe the direction of the effect.	8-15
	20c	Present results of all investigations of possible causes of heterogeneity among study results.	8-15
	20d	Present results of all sensitivity analyses conducted to assess the robustness of the synthesized results.	8-15
Reporting biases	21	Present assessments of risk of bias due to missing results (arising from reporting biases) for each synthesis assessed.	8
Certainty of evidence	22	Present assessments of certainty (or confidence) in the body of evidence for each outcome assessed.	8
**DISCUSSION**			
Discussion	23a	Provide a general interpretation of the results in the context of other evidence.	15
	23b	Discuss any limitations of the evidence included in the review.	16-17

Section and Topic	Item #	Checklist item	Location where item is reported
	23c	Discuss any limitations of the review processes used.	16-17
	23d	Discuss implications of the results for practice, policy, and future research.	17
**OTHER INFORMATION**			
Registration and protocol	24a	Provide registration information for the review, including register name and registration number, or state that the review was not registered.	2
	24b	Indicate where the review protocol can be accessed, or state that a protocol was not prepared.	2
	24c	Describe and explain any amendments to information provided at registration or in the protocol.	2
Support	25	Describe sources of financial or non-financial support for the review, and the role of the funders or sponsors in the review.	1
Competing interests	26	Declare any competing interests of review authors.	17
Availability of data, code and other materials	27	Report which of the following are publicly available and where they can be found: template data collection forms; data extracted from included studies; data used for all analyses; analytic code; any other materials used in the review.	18

From: Page MJ, McKenzie JE, Bossuyt PM, Boutron I, Hoffmann TC, Mulrow CD, et al. The PRISMA 2020 statement: an updated guideline for reporting systematic reviews. BMJ 2021;372:n71. doi: 10.1136/bmj.n71

For more information, visit: http://www.prisma-statement.org/

Appendix B. Enhancing transparency in reporting the synthesis of qualitative research: the ENTREQ statement checklist

No	Item	Guide and description	Page
1	Aim	State the research question the synthesis addresses.	3
2	Synthesis methodology	Identify the synthesis methodology or theoretical framework which underpins the synthesis, and describe the rationale for choice of methodology *(e.g. meta-ethnography, thematic synthesis, critical interpretive synthesis, grounded theory synthesis, realist synthesis, meta-aggregation, meta-study, framework synthesis).*	6
3	Approach to searching	Indicate whether the search was pre-planned (*comprehensive search strategies to seek all available studies)* or iterative (*to seek all available concepts until they theoretical saturation is achieved)*.	4
4	Inclusion criteria	Specify the inclusion/exclusion criteria *(e.g. in terms of population, language, year limits, type of publication, study type).*	4-5
5	Data sources	Describe the information sources used (e.g. *electronic databases (MEDLINE, EMBASE, CINAHL, psycINFO, Econlit), grey literature databases (digital thesis, policy reports), relevant organisational websites, experts, information specialists, generic web searches (Google Scholar) hand searching, reference lists)* and when the searches conducted; provide the rationale for using the data sources.	4
6	Electronic Search strategy	Describe the literature search *(e.g. provide electronic search strategies with population terms, clinical or health topic terms, experiential or social phenomena related terms, filters for qualitative research, and search limits)*.	4
7	Study screening methods	Describe the process of study screening and sifting *(e.g. title, abstract and full text review, number of independent reviewers who screened studies).*	5
8	Study characteristics	Present the characteristics of the included studies *(e.g. year of publication, country, population, number of participants, data collection, methodology, analysis, research questions).*	7-8
9	Study selection results	Identify the number of studies screened and provide reasons for study exclusion *(e,g, for comprehensive searching, provide numbers of studies screened and reasons for exclusion indicated in a figure/flowchart; for iterative searching describe reasons for study exclusion and inclusion based on modifications t the research question and/or contribution to*	7

No	Item	Guide and description	Page
		theory development).	
10	Rationale for appraisal	Describe the rationale and approach used to appraise the included studies or selected findings *(e.g. assessment of conduct (validity and robustness), assessment of reporting (transparency), assessment of content and utility of the findings).*	8
11	Appraisal items	State the tools, frameworks and criteria used to appraise the studies or selected findings *(e.g. Existing tools: CASP, QARI, COREQ, Mays and Pope* [25]*; reviewer developed tools; describe the domains assessed: research team, study design, data analysis and interpretations, reporting).*	8
12	Appraisal process	Indicate whether the appraisal was conducted independently by more than one reviewer and if consensus was required.	6
13	Appraisal results	Present results of the quality assessment and indicate which articles, if any, were weighted/excluded based on the assessment and give the rationale.	8
14	Data extraction	Indicate which sections of the primary studies were analysed and how were the data extracted from the primary studies? *(e.g. all text under the headings "results /conclusions" were extracted electronically and entered into a computer software).*	5-6
15	Software	State the computer software used, if any.	5
16	Number of reviewers	Identify who was involved in coding and analysis.	5
17	Coding	Describe the process for coding of data *(e.g. line by line coding to search for concepts).*	6
18	Study comparison	Describe how were comparisons made within and across studies *(e.g. subsequent studies were coded into pre-existing concepts, and new concepts were created when deemed necessary).*	6
19	Derivation of themes	Explain whether the process of deriving the themes or constructs was inductive or deductive.	6
20	Quotations	Provide quotations from the primary studies to illustrate themes/constructs, and identify whether the quotations were participant quotations of the author's interpretation.	8-15
21	Synthesis output	Present rich, compelling and useful results that go beyond a summary of the primary studies (e.g. *new interpretation,*	8-15

No	Item	Guide and description	Page
		models of evidence, conceptual models, analytical framework, development of a new theory or construct).	

Appendix C. Search terms and search strategies

ID	Query	Items found
	**Medline (Inception to 17th Nov 2023)**	
1	Fibromyalgia/	10918
2	fibromyalgia.ab,kf,ti.	14450
3	fibromyositis.ab,kf,ti.	24
4	fibrositis.ab,kf,ti.	606
5	myofascial pain.ab,kf,ti.	3133
6	chronic generalized pain.ab,kf,ti.	30
7	chronic generalised pain.ab,kf,ti.	1
8	chronic widespread pain.ab,kf,ti.	1044
9	1 or 2 or 3 or 4 or 5 or 6 or 7 or 8	19185
10	exp Self Care/	66290
11	(self adj2 (admin$ or care or efficacy or guid$ or help or improve$ or manag$ or monitor$)).ab,kf,ti.	203018
12	Patient Education as Topic/	88811
13	((patient$ or adult$ or client$ or participant$ or individual$) adj2 (train$ or educat$ or teach$ or instruct$ or inform$ or counsel$ or empower$ or advic$ or advis$)).ab,kf,ti.	215185
14	expert patient$.ab,kf,ti.	401
15	Psychoeducation$.ab,kf,ti.	8793
16	(bibliotherapy or book$ or leaflet$ or CD or compact disc$ or learn$ or guid$ or facilitat$).ab,kf,ti.	2971362
17	exp Telecommunications/	138786
18	(telemedicine or tele medicine).ab,kf,ti.	33346
19	(telehealth or tele health or tele-health).ab,kf,ti.	21187
20	(ehealth or e-health).ab,kf,ti.	14413
21	(mobile health or mhealth or m-health).ab,kf,ti.	19331
22	ICT.ab,kf,ti.	9823
23	((inform$ or communicat$ or interact$) adj6 (computer$ or technolog$ or software)).ab,kf,ti.	74368
24	((health$ or treat$ or therap$ or intervention$ or assist$ or selfmanag$ or self-manag$) adj6 (computer$ or technolog$ or software)).ab,kf,ti.	168804
25	(interactive voice response or IVR).ab,kf,ti.	2887
26	exp Internet/	107095
27	(internet$ or world wide web or www or web or web-based or email$ or e-mail$ or online).ab,kf,ti.	603074
28	(telephone$ or phone$ or mobile$ or cellphone$ or app or apps or text$ or SMS or smartphone$).ab,kf,ti.	593295
29	(virtual reality or augmented reality or VR or AR).ab,kf,ti.	103856
30	lay-led.ab,kf,ti.	66
31	peer-led.ab,kf,ti.	1922
32	professionally-led.ab,kf,ti.	140
33	therapist-led.ab,kf,ti.	298
34	psychologist-led.ab,kf,ti.	37
35	physiotherapist-led.ab,kf,ti.	207
36	rheumatologist-led.ab,kf,ti.	17

37	Rehabilitation.ab,kf,ti.	246835
38	Mindfulness*.ab,kf,ti.	17319
39	(Acceptance and commitment therapy).ab,kf,ti.	2361
40	(exerci$ adj2 (train$ or educat$ or teach$ or instruct$ or inform$ or counsel$ or empower$ or advic$ or advis$)).ab,kf,ti.	36438
41	(self adj2 hydrotherapy).ab,kf,ti.	3
42	(Pain management adj2 (train$ or educat$ or teach$ or instruct$ or inform$ or counsel$ or empower$ or advic$ or advis$)).ab,kf,ti.	1034
43	exp Cognitive Behavioral Therapy/	41114
44	(fatigue adj2 (train$ or educat$ or teach$ or instruct$ or inform$ or counsel$ or empower$ or advic$ or advis$)).ab,kf,ti.	912
45	((nutrition or food or diet) adj2 (train$ or educat$ or teach$ or instruct$ or inform$ or counsel$ or empower$ or advic$ or advis$)).ab,kf,ti.	20764
46	(sleep adj2 (train$ or educat$ or teach$ or instruct$ or inform$ or counsel$ or empower$ or advic$ or advis$)).ab,kf,ti.	3277
47	Physical Therapy Specialty/ or exp Exercise Therapy/	73112
48	(Chronic disease self-management program or CDSMP or (Stanford model and Chronic Disease)).ti,ab,kw.	235
49	10 or 11 or 12 or 13 or 14 or 15 or 16 or 17 or 18 or 19 or 20 or 21 or 22 or 23 or 24 or 25 or 26 or 27 or 28 or 29 or 30 or 31 or 32 or 33 or 34 or 35 or 36 or 37 or 38 or 39 or 40 or 41 or 42 or 43 or 44 or 45 or 46 or 47 or 48	4799878
50	9 and 49	4597
51	Qualitative Research/ or interviews as topic/ or focus groups/ or narration/ or questionnaires/ or self report/ or exp attitudes/ or exp tape recording/ or Nursing Methodology Research/	1292673
52	(qualitative or ethno$ or emic or etic or phenomenolog$ or hermeneutic$ or Heidegger$ or Husserl$ or Colazzi$ or Giorgi$ or Glaser$ or Strauss$ or Van Kaam$ or Van Manen$).mp.	662537
53	(constant compar$ or focus group$ or grounded theory or narrative analysis or lived experience$ or life experience$ or theoretical sampl$ or purposive sampl$ or ricoeur$ or speigelberg$ or merleau$ or metasynthes$ or meta-synthes$ or metasummar$ or metasummar$ or metastud$ or meta-stud$ or maximum variation or snowball$ or field stud$ or field note$ or fieldnote$ or field record$ or content analy$ or unstructured categor$ or structured categor$ or action research or audiorecord$ or taperecord$ or videorecord$ or videotap$ or digitalrecord$ or digitaltap$).mp.	251465
54	(thematic$ adj3 analy$).mp.	82509
55	((participant$ or nonparticipant$ or non-participant$ or non participant$) adj3 observ$).mp.	18653
56	((audio or tape or tapes or taping or video$ or digital$) adj5 (record$ or interview$)).mp.	91789
57	interview.tw.	192374
58	51 or 52 or 53 or 54 or 55 or 56 or 57	2026844
59	50 and 58	1147
	**EMBASE**	
1	exp fibromyalgia/	29032
2	fibromyalgia.ab,kw,ti.	23672
3	fibromyositis.ab,kw,ti.	49
4	fibrositis.ab,kw,ti.	698
5	myofascial pain.ab,kw,ti.	3988
6	chronic generalized pain.ab,kw,ti.	44
7	chronic generalised pain.ab,kw,ti.	3
8	chronic widespread pain.ab,kw,ti.	1693

9	1 or 2 or 3 or 4 or 5 or 6 or 7 or 8	35674
10	exp Self-Care/	117832
11	(self adj2 (admin$ or care or efficacy or guid$ or help or improve$ or manag$ or monitor$)).ab,kw,ti.	256070
12	patient education/	133609
13	((patient$ or adult$ or client$ or participant$ or individual$) adj2 (train$ or educat$ or teach$ or instruct$ or inform$ or counsel$ or empower$ or advic$ or advis$)).ab,kw,ti.	339949
14	expert patient$.ab,kw,ti.	700
15	Psychoeducation$.ab,kw,ti.	12528
16	(bibliotherapy or book$ or leaflet$ or CD or compact disc$ or DVD or learn$ or guid$ or facilitat$).ab,kw,ti.	3906206
17	exp telecommunication/	145471
18	(telemedicine or tele medicine).ab,kw,ti.	42595
19	(telehealth or tele health or tele-health).ab,kw,ti.	25983
20	(ehealth or e-health).ab,kw,ti.	14948
21	(mobile health or mhealth or m-health).ab,kw,ti.	18003
22	ICT.ab,kw,ti.	12292
23	((inform$ or communicat$ or interact$) adj6 (computer$ or technolog$ or software)).ab,kw,ti.	85590
24	((health$ or treat$ or therap$ or intervention$ or assist$ or selfmanag$ or self-manag$) adj6 (computer$ or technolog$ or software)).ab,kw,ti.	210589
25	(interactive voice response or IVR).ab,kw,ti.	3843
26	exp Internet/	142044
27	(internet$ or world wide web or www or web or web-based or email$ or e-mail$ or online).ab,kw,ti.	754787
28	(telephone$ or phone$ or mobile$ or cellphone$ or app or apps or text$ or SMS or smartphone$).ab,kw,ti.	747368
29	(virtual reality or augmented reality or VR or AR).ab,kw,ti.	137773
30	lay-led.ab,kw,ti.	80
31	peer-led.ab,kw,ti.	2452
32	professionally-led.ab,kw,ti.	220
33	therapist-led.ab,kw,ti.	423
34	psychologist-led.ab,kw,ti.	54
35	physiotherapist-led.ab,kw,ti.	353
36	rheumatologist-led.ab,kw,ti.	47
37	Rehabilitation.ab,kw,ti.	348854
38	Mindfulness*.ab,kw,ti.	20899
39	(acceptance and commitment therapy).ab,kw,ti.	2832
40	(exerci$ adj2 (train$ or educat$ or teach$ or inform$ or counsel$ or empower$ or advic$ or advis$)).ab,kw,ti.	49334
41	(self adj2 hydrotherapy).ab,kw,ti.	6
42	(Pain management adj2 (train$ or educat$ or teach$ or instruct$ or inform$ or counsel$ or empower$ or advic$ or advis$)).ab,kw,ti.	1632
43	exp cognitive therapy/ or exp Behaviour Therapy/	124769
44	(fatigue adj2 (train$ or educat$ or teach$ or instruct$ or inform$ or counsel$ or empower$ or advic$ or advis$)).ab,kw,ti.	1466
45	((nutrition or food or diet) adj2 (train$ or educat$ or teach$ or instruct$ or inform$ or counsel$ or empower$ or advic$ or advis$)).ab,kw,ti.	29219
46	((nutrition or food or diet) adj2 (train$ or educat$ or teach$ or instruct$ or inform$ or	5426

	counsel$ or empower$ or advic$ or advis$)).ab,kw,ti.	
47	(Chronic disease self-management program or CDSMP or (Stanford model and Chronic Disease)).ti,ab,kw.	299
48	exp physiotherapy practice/ or exp physiotherapy/	133902
49	10 or 11 or 12 or 13 or 14 or 15 or 16 or 17 or 18 or 19 or 20 or 21 or 22 or 23 or 24 or 25 or 26 or 27 or 28 or 29 or 30 or 31 or 32 or 33 or 34 or 35 or 36 or 37 or 38 or 39 or 40 or 41 or 42 or 43 or 44 or 45 or 46 or 47 or 48	6335877
50	9 and 49	10003
51	exp qualitative research/ or exp interview/ or focus groups/ or verbal communication/ or questionnaire/ or self report/ or exp attitude/ or exp recording/ or nursing methodology/	2781384
52	(qualitative or ethno$ or emic or etic or phenomenolog$ or hermeneutic$ or heidegger$ or husserl$ or colaizzi$ or giorgi$ or glaser$ or strauss$ or van kaam$ or van manen$).ab,kw,ti.	572271
53	(constant compar$ or focus group$ or grounded theory or narrative analysis or lived experience$ or life experience$ or theoretical sampl$ or purposive sampl$ or ricoeur$ or spiegelberg$ or merleau$ or metasynthes$ or meta-synthes$ or metasummar$ or meta-summar$ or metastud$ or maximum variation or snowball$ or field stud$ or field note$ or fieldnote$ or field recORd$ or content analy$ or unstructured categor$ or unstructured categor$ or structured categor$ or action research or audiorecord$ or taperecord$ or videorecord$ or videotap$ or digitalrecord$ or digitaltap$).ab,kw,ti.	282881
54	(thematic$ adj3 analy$).ab,kw,ti.	95992
55	((participant$ or nonparticipant$ or non-participant$) adj3 observ$).ab,kw,ti.	23968
56	((audio or tape or taping or video$ or digital$) adj5 (record$ or interview$)).ab,kw,ti.	75066
57	interview.tw.	252010
58	51 or 52 or 53 or 54 or 55 or 56 or 57	3304954
59	50 and 58	3068
**PsycINFO**		
S58	S49 AND S57	382
S57	S50 OR S51 OR S52 OR S53 OR S54 OR S55 OR S56	1177694
S56	DE Qualitative research OR DE interviews as topics OR DE focus groups OR DE narration OR DE questionnaires OR DE self report OR DE attitudes OR DE tape recording OR DE nursing methodology research	101240
S55	TI (qualitative OR ethno* OR emic OR etic OR phenomenolog* OR hermeneutic* OR heidegger* OR Husserl* OR colaizzi* OR giorgi* OR glaser* OR strauss* OR "van kaam*" OR "van manen*") OR AB (qualitative OR ethno* OR emic OR etic OR phenomenolog* OR hermeneutic* OR heidegger* OR Husserl* OR colaizzi* OR giorgi* OR glaser* OR strauss* OR "van kaam*" OR "van manen*") OR KW (qualitative OR ethno* OR emic OR etic OR phenomenolog* OR hermeneutic* OR heidegger* OR Husserl* OR colaizzi* OR giorgi* OR glaser* OR strauss* OR "van kaam*" OR "van manen*")	297376

S54	TI (constant compar* OR focus group* OR grounded theory OR narrative analysis or lived experience* OR life experience* OR theoretical sampl* OR purposive sampl* OR ricoeur* OR speigelberg* OR merleau* OR metasynthes* OR meta-synthes* OR metasummar* OR meta-summar* OR metastud* OR meta-stud* OR maximum variation OR snowball* OR field stud* OR field note* OR fieldnote* OR field record* OR content analy* OR unstructured categor* OR structured categor* OR action research OR audiorecord* OR taperecord* OR videorecord* OR videotap* OR digitalrecord* OR digitaltap*) OR AB (constant compar* OR focus group* OR grounded theory OR narrative analysis or lived experience* OR life experience* OR theoretical sampl* OR purposive sampl* OR ricoeur* OR speigelberg* OR merleau* OR metasynthes* OR meta-synthes* OR metasummar* OR meta-summar* OR metastud* OR meta-stud* OR maximum variation OR snowball* OR field stud* OR field note* OR fieldnote* OR field record* OR content analy* OR unstructured categor* OR structured categor* OR action research OR audiorecord* OR taperecord* OR videorecord* OR videotap* OR digitalrecord* OR digitaltap*) OR KW (constant compar* OR focus group* OR grounded theory OR narrative analysis or lived experience* OR life experience* OR theoretical sampl* OR purposive sampl* OR ricoeur* OR speigelberg* OR merleau* OR metasynthes* OR meta-synthes* OR metasummar* OR meta-summar* OR metastud* OR meta-stud* OR maximum variation OR snowball* OR field stud* OR field note* OR fieldnote* OR field record* OR content analy* OR unstructured categor* OR structured categor* OR action research OR audiorecord* OR taperecord* OR videorecord* OR videotap* OR digitalrecord* OR digitaltap*)	732037
S53	TI thematic* N3 analy* OR AB thematic* N3 analy* OR KW thematic* N3 analy*	34581
S52	TI ((participant* OR nonparticipant* OR non-participant*) N3 observ*) OR AB ((participant* OR nonparticipant* OR non-participant*) N3 observ*) OR KW ((participant* OR nonparticipant* OR non-participant*) N3 observ*)	17771
S51	TI ((audio OR tape OR tapes OR taping OR video* OR digital*) N5 (record* OR interview*)) OR AB ((audio OR tape OR tapes OR taping OR video* OR digital*) N5 (record* OR interview*)) OR KW ((audio OR tape OR tapes OR taping OR video* OR digital*) N5 (record* OR interview*))	31285
S50	TI interview* OR AB interview* OR KW interview*	388429
S49	S9 AND S48	1560
S48	S10 OR S11 OR S12 OR S13 OR S14 OR S15 OR S16 OR S17 OR S18 OR S19 OR S20 OR S21 OR S22 OR S23 OR S24 OR S25 OR S26 OR S27 OR S28 OR S29 OR S30 OR S31 OR S32 OR S33 OR S34 OR S35 OR S36 OR S37 OR S38 OR S39 OR S40 OR S41 OR S42 OR S43 OR S44 OR S45 OR S46 OR S47	1683192
S47	TI (exerci* N2 (train* OR educat* OR teach* OR instruct* OR inform* OR counsel* OR empower* OR advic* OR advis*)) OR AB (exerci* N2 (train* OR educat* OR teach* OR instruct* OR inform* OR counsel* OR empower* OR advic* OR advis*)) OR KW (exerci* N2 (train* OR educat* OR teach* OR instruct* OR inform* OR counsel* OR empower* OR advic* OR advis*))	6461
S46	TI (Fatigue N2 (train* OR educat* OR teach* OR instruct* OR inform* OR counsel* OR empower* OR advic* OR advis*)) OR AB (Fatigue N2 (train* OR educat* OR teach* OR instruct* OR inform* OR counsel* OR empower* OR advic* OR advis*)) OR KW (Fatigue N2 (train* OR educat* OR teach* OR instruct* OR inform* OR counsel* OR empower* OR advic* OR advis*))	495
S45	TI ((pain management) N2 (train* OR educat* OR teach* OR instruct* OR inform* OR counsel* OR empower* OR advic* OR advis*)) OR AB ((pain management) N2 (train* OR educat* OR teach* OR instruct* OR inform* OR counsel* OR empower* OR advic* OR advis*)) OR KW ((pain management) N2 (train* OR educat* OR teach* OR instruct* OR inform* OR counsel* OR empower* OR advic* OR advis*))	1265
S44	DE Cognitive Therapy OR DE Cognitive Behavio*r Therapy OR DE Behavio*r Therapy	67376

S43	TI ((nutrition OR food OR diet) N2 (train* OR educat* OR teach* OR instruct* OR inform* OR counsel* OR empower* OR advic* OR advis*)) OR AB ((nutrition OR food OR diet) N2 (train* OR educat* OR teach* OR instruct* OR inform* OR counsel* OR empower* OR advic* OR advis*)) OR KW ((nutrition OR food OR diet) N2 (train* OR educat* OR teach* OR instruct* OR inform* OR counsel* OR empower* OR advic* OR advis*))	6419
S42	TI (Sleep N2 (train* OR educat* OR teach* OR instruct* OR inform* OR counsel* OR empower* OR advic* OR advis*)) OR AB (Sleep N2 (train* OR educat* OR teach* OR instruct* OR inform* OR counsel* OR empower* OR advic* OR advis*)) OR KW (Sleep N2 (train* OR educat* OR teach* OR instruct* OR inform* OR counsel* OR empower* OR advic* OR advis*))	1946
S41	TI (chronic disease self-management program OR CDSMP OR (Stanford Model AND Chronic Disease)) OR AB (chronic disease self-management program OR CDSMP OR (Stanford Model AND Chronic Disease)) OR KW (chronic disease self-management program OR CDSMP OR (Stanford Model AND Chronic Disease))	535
S40	DE Physical Therapy specialty OR DE Exercise Therapy	3814
S39	TI ((Acceptance and commitment therapy)) OR AB ((Acceptance and commitment therapy)) OR KW ((Acceptance and commitment therapy))	3213
S38	TI Mindfulness OR AB Mindfulness OR KW Mindfulness	19094
S37	TI rehabilitation OR AB rehabilitation OR KW rehabilitation	67074
S36	TI rheumatologist-led OR AB rheumatologist-led OR KW rheumatologist-led	5
S35	TI physiotherapist-led OR AB physiotherapist-led OR KW physiotherapist-led	21
S34	TI psychologist-led OR AB psychologist-led OR KW psychologist-led	36
S33	TI therapist-led OR AB therapist-led OR KW therapist-led	168
S32	TI professionally-led OR AB professionally-led OR KW professionally-led	130
S31	TI peer-led OR AB peer-led OR KW peer-led	1041
S30	TI lay-led OR AB lay-led OR KW lay-led	35
S29	TI (virtual reality OR augmented reality OR VR OR AR) OR AB (virtual reality OR augmented reality OR VR OR AR) OR KW (virtual reality OR augmented reality OR VR OR AR)	14904
S28	TI (telephone* OR phone* OR mobile* OR cellphone* OR app OR apps OR text* OR SMS OR smartphone*) OR AB (telephone* OR phone* OR mobile* OR cellphone* OR app OR apps OR text* OR SMS OR smartphone*) OR KW (telephone* OR phone* OR mobile* OR cellphone* OR app OR apps OR text* OR SMS OR smartphone*)	206093
S27	TI (internet* OR world wide web OR www OR web OR web-based OR email* OR e-mail* OR online) OR AB (internet* OR world wide web OR www OR web OR web-based OR email* OR e-mail* OR online) OR KW (internet* OR world wide web OR www OR web OR web-based OR email* OR e-mail* OR online)	192414
S26	DE internet	38282
S25	TI (interactive voice response OR IVR) OR AB (interactive voice response OR IVR) OR KW (interactive voice response OR IVR)	635
S24	TI ((health* OR treat* OR therap* OR intervention* OR assist* OR selfmanag* OR self-manag*) N6 (computer* OR technolog* OR software)) OR AB ((health* OR treat* OR therap* OR intervention* OR assist* OR selfmanag* OR self-manag*) N6 (computer* OR technolog* OR software)) OR KW ((health* OR treat* OR therap* OR intervention* OR assist* OR selfmanag* OR self-manag*) N6 (computer* OR technolog* OR software))	32097
S23	TI ((inform* OR communicat* OR interact*) N6 (computer* OR technolog* OR software)) OR AB ((inform* OR communicat* OR interact*) N6 (computer* OR technolog* OR software)) OR KW ((inform* OR communicat* OR interact*) N6 (computer* OR technolog* OR software))	42620
S22	TI ICT OR AB ICT OR KW ICT	5076

S21	TI (mobile health OR mhealth OR m-health) OR AB (mobile health OR mhealth OR m-health) OR KW (mobile health OR mhealth OR m-health)	6286
S20	TI (ehealth OR e-health) OR AB (ehealth OR e-health) OR KW (ehealth OR e-health)	2769
S19	TI (telehealth OR tele health OR tele-health) OR AB (telehealth OR tele health OR tele-health) OR KW (telehealth OR tele health OR tele-health)	4177
S18	TI (telemedicine OR tele medicine) OR AB (telemedicine OR tele medicine) OR KW (telemedicine OR tele medicine)	3288
S17	DE telecommunications	337
S16	TI (bibliotherapy OR book* OR cd OR compact disc* OR dvd OR learn* OR guid* OR facilitat*) OR AB (bibliotherapy OR book* OR cd OR compact disc* OR dvd OR learn* OR guid* OR facilitat*) OR KW (bibliotherapy OR book* OR cd OR compact disc* OR dvd OR learn* OR guid* OR facilitat*)	1109979
S15	TI Psychoeducation* OR AB Psychoeducation* OR KW Psychoeducation*	11573
S14	TI expert patient* OR AB expert patient* OR KW expert patient*	11442
S13	TI ((patient* OR adult* OR client* OR participant* OR individual*) N2 (train* OR educat* OR teach* OR instruct* OR inform* OR counsel* OR empower* OR advic* OR advis*)) OR AB ((patient* OR adult* OR client* OR participant* OR individual*) N2 (train* OR educat* OR teach* OR instruct* OR inform* OR counsel* OR empower* OR advic* OR advis*)) OR KW ((patient* OR adult* OR client* OR participant* OR individual*) N2 (train* OR educat* OR teach* OR instruct* OR inform* OR counsel* OR empower* OR advic* OR advis*))	117562
S12	DE education	46592
S11	TI (Self N2 (admin* OR care OR efficac* OR guid* OR help OR improve* OR manag* OR monitor*)) OR AB (Self N2 (admin* OR care OR efficac* OR guid* OR help OR improve* OR manag* OR monitor*)) OR KW (Self N2 (admin* OR care OR efficac* OR guid* OR help OR improve* OR manag* OR monitor*))	126067
S10	DE self care	7622
S9	S1 OR S2 OR S3 OR S4 OR S5 OR S6 OR S7 OR S8	5247
S8	TI chronic widespread pain OR AB chronic widespread pain OR KW chronic widespread pain	791
S7	TI chronic generalised pain OR AB chronic generalised pain OR KW chronic generalised pain	30
S6	TI chronic generalized pain OR AB chronic generalized pain OR KW chronic generalized pain	467
S5	TI myofascial pain OR AB myofascial pain OR KW myofascial pain	586
S4	TI fibrositis OR AB fibrositis OR KW fibrositis	40
S3	TI fibromyositis OR AB fibromyositis OR KW fibromyositis	4
S2	TI fibromyalgia OR AB fibromyalgia OR KW fibromyalgia	3780
S1	DE Fibromyalgia	2749
**CINAHL**		
S58	S49 AND S57	673
S57	S50 OR S51 OR S52 OR S53 OR S54 OR S55 OR S56	929780
S56	MH Qualitative Studies OR MH interviews OR MH focus groups OR MH narratives OR MH surveys OR MH self report OR MH attitude OR MH audiorecording OR MH nursing methodology research	534064
S55	TI (qualitative OR ethno* OR emic OR etic OR phenomenolog* OR hermeneutic* OR heidegger* OR Husserl* OR colaizzi* OR giorgi* OR glaser* OR strauss* OR "van kaam*" OR "van manen*") OR AB (qualitative OR ethno* OR emic OR etic OR phenomenolog* OR hermeneutic* OR heidegger* OR Husserl* OR colaizzi* OR giorgi* OR glaser* OR strauss* OR "van kaam*" OR "van manen*")	209974

S54	TI (constant compar* OR focus group* OR grounded theory OR narrative analysis or lived experience* OR life experience* OR theoretical sampl* OR purposive sampl* OR ricoeur* OR speigelberg* OR merleau* OR metasynthes* OR meta-synthes* OR metasummar* OR meta-summar* OR metastud* OR meta-stud* OR maximum variation OR snowball* OR field stud* OR field note* OR fieldnote* OR field record* OR content analy* OR unstructured categor* OR structured categor* OR action research OR audiorecord* OR taperecord* OR videorecord* OR videotap* OR digitalrecord* OR digitaltap*) OR AB (constant compar* OR focus group* OR grounded theory OR narrative analysis or lived experience* OR life experience* OR theoretical sampl* OR purposive sampl* OR ricoeur* OR speigelberg* OR merleau* OR metasynthes* OR meta-synthes* OR metasummar* OR meta-summar* OR metastud* OR meta-stud* OR maximum variation OR snowball* OR field stud* OR field note* OR fieldnote* OR field record* OR content analy* OR unstructured categor* OR structured categor* OR action research OR audiorecord* OR taperecord* OR videorecord* OR videotap* OR digitalrecord* OR digitaltap*)	371990
S53	TI thematic* N3 analy* OR AB thematic* N3 analy*	43200
S52	TI ((participant* OR nonparticipant* OR non-participant*) N3 observ*) OR AB ((participant* OR nonparticipant* OR non-participant*) N3 observ*)	11850
S51	TI ((audio OR tape OR tapes OR taping OR video* OR digital*) N5 (record* OR interview*)) OR AB ((audio OR tape OR tapes OR taping OR video* OR digital*) N5 (record* OR interview*))	20704
S50	TI interview* OR AB interview*	272213
S49	S9 AND S48	3252
S48	S10 OR S11 OR S12 OR S13 OR S14 OR S15 OR S16 OR S17 OR S18 OR S19 OR S20 OR S21 OR S22 OR S23 OR S24 OR S25 OR S26 OR S27 OR S28 OR S29 OR S30 OR S31 OR S32 OR S33 OR S34 OR S35 OR S36 OR S37 OR S38 OR S39 OR S40 OR S41 OR S42 OR S43 OR S44 OR S45 OR S46 OR S47	1501408
S47	TI (exerci* N2 (train* OR educat* OR teach* OR instruct* OR inform* OR counsel* OR empower* OR advic* OR advis*)) OR AB (exerci* N2 (train* OR educat* OR teach* OR instruct* OR inform* OR counsel* OR empower* OR advic* OR advis*))	15933
S46	TI (Fatigue N2 (train* OR educat* OR teach* OR instruct* OR inform* OR counsel* OR empower* OR advic* OR advis*)) OR AB (Fatigue N2 (train* OR educat* OR teach* OR instruct* OR inform* OR counsel* OR empower* OR advic* OR advis*))	747
S45	TI ((pain management) N2 (train* OR educat* OR teach* OR instruct* OR inform* OR counsel* OR empower* OR advic* OR advis*)) OR AB ((pain management) N2 (train* OR educat* OR teach* OR instruct* OR inform* OR counsel* OR empower* OR advic* OR advis*))	3016
S44	MH Cognitive Therapy OR MH Behavior Therapy	34549
S43	TI ((nutrition OR food OR diet) N2 (train* OR educat* OR teach* OR instruct* OR inform* OR counsel* OR empower* OR advic* OR advis*)) OR AB ((nutrition OR food OR diet) N2 (train* OR educat* OR teach* OR instruct* OR inform* OR counsel* OR empower* OR advic* OR advis*))	14985
S42	TI (Sleep N2 (train* OR educat* OR teach* OR instruct* OR inform* OR counsel* OR empower* OR advic* OR advis*)) OR AB (Sleep N2 (train* OR educat* OR teach* OR instruct* OR inform* OR counsel* OR empower* OR advic* OR advis*))	1867
S41	TI (chronic disease self-management program OR CDSMP OR (Stanford Model AND Chronic Disease)) OR AB (chronic disease self-management program OR CDSMP OR (Stanford Model AND Chronic Disease))	1073
S40	MH Physical Therapy OR MH Therapeutic Exercise	65204
S39	TI ((Acceptance and commitment therapy)) OR AB ((Acceptance and commitment therapy))	1189
S38	TI Mindfulness OR AB Mindfulness	9116
S37	TI rehabilitation OR AB rehabilitation	109207

S36	TI rheumatologist-led OR AB rheumatologist-led	9
S35	TI physiotherapist-led OR AB physiotherapist-led	132
S34	TI psychologist-led OR AB psychologist-led	13
S33	TI therapist-led OR AB therapist-led	168
S32	TI professionally-led OR AB professionally-led	101
S31	TI peer-led OR AB peer-led	1039
S30	TI lay-led OR AB lay-led	59
S29	TI (virtual reality OR augmented reality OR VR OR AR) OR AB (virtual reality OR augmented reality OR VR OR AR)	14690
S28	TI (telephone* OR phone* OR mobile* OR cellphone* OR app OR apps OR text* OR SMS OR smartphone*) OR AB (telephone* OR phone* OR mobile* OR cellphone* OR app OR apps OR text* OR SMS OR smartphone*)	147891
S27	TI (internet* OR world wide web OR www OR web OR web-based OR email* OR e-mail* OR online) OR AB (internet* OR world wide web OR www OR web OR web-based OR email* OR e-mail* OR online)	206984
S26	MH internet	55536
S25	TI (interactive voice response OR IVR) OR AB (interactive voice response OR IVR)	923
S24	TI ((health* OR treat* OR therap* OR intervention* OR assist* OR selfmanag* OR self-manag*) N6 (computer* OR technolog* OR software)) OR AB ((health* OR treat* OR therap* OR intervention* OR assist* OR selfmanag* OR self-manag*) N6 (computer* OR technolog* OR software))	55635
S23	TI ((inform* OR communicat* OR interact*) N6 (computer* OR technolog* OR software)) OR AB ((inform* OR communicat* OR interact*) N6 (computer* OR technolog* OR software))	35317
S22	TI ICT OR AB ICT	2523
S21	TI (mobile health OR mhealth OR m-health) OR AB (mobile health OR mhealth OR m-health)	11702
S20	TI (ehealth OR e-health) OR AB (ehealth OR e-health)	4697
S19	TI (telehealth OR tele health OR tele-health) OR AB (telehealth OR tele health OR tele-health)	9199
S18	TI (telemedicine OR tele medicine) OR AB (telemedicine OR tele medicine)	8751
S17	MH telecommunications	2723
S16	TI (bibliotherapy OR book* OR cd OR compact disc* OR dvd OR learn* OR guid* OR facilitat*) OR AB (bibliotherapy OR book* OR cd OR compact disc* OR dvd OR learn* OR guid* OR facilitat*)	787390
S15	TI Psychoeducation* OR AB Psychoeducation*	3970
S14	TI expert patient* OR AB expert patient*	30459
S13	TI ((patient* OR adult* OR client* OR participant* OR individual*) N2 (train* OR educat* OR teach* OR instruct* OR inform* OR counsel* OR empower* OR advic* OR advis*)) OR AB ((patient* OR adult* OR client* OR participant* OR individual*) N2 (train* OR educat* OR teach* OR instruct* OR inform* OR counsel* OR empower* OR advic* OR advis*))	132812
S12	MH education	11863
S11	TI (Self N2 (admin* OR care OR efficac* OR guid* OR help OR improve* OR manag* OR monitor*)) OR AB (Self N2 (admin* OR care OR efficac* OR guid* OR help OR improve* OR manag* OR monitor*))	102353
S10	MH self care	46396
S9	S1 OR S2 OR S3 OR S4 OR S5 OR S6 OR S7 OR S8	12141
S8	TI chronic widespread pain OR AB chronic widespread pain	1278

S7	TI chronic generalised pain OR AB chronic generalised pain	70
S6	TI chronic generalized pain OR AB chronic generalized pain	579
S5	TI myofascial pain OR AB myofascial pain	2585
S4	TI fibrositis OR AB fibrositis	28
S3	TI fibromyositis OR AB fibromyositis	2
S2	TI fibromyalgia OR AB fibromyalgia	6811
S1	MH Fibromyalgia	6524
	**Web of Science**	
#1	TS=(Fibromyalgia OR Fibromyositis OR Fibrositis)	21301
#2	TS=(myofascial pain OR Chronic generalized pain OR chronic generalised pain OR chronic widespread pain)	11569
#3	#2 OR #1	30072
#4	TS=(self NEAR/2 (admin* OR care OR efficacy OR guid* OR help OR improve* OR manag* OR monitor*))	272751
#5	TS=((patient* OR adult* OR client* OR participant* OR individual*) NEAR/2 (train* OR educat* OR teach* OR instruct* OR inform* OR counsel* OR empower* OR advic* OR advis*))	308397
#6	TS=(expert patient*)	92553
#7	TS=(psychoeducation*)	10465
#8	TS=(bibliotherapy OR book* OR leaflet* OR CD OR compact disc* OR DVD OR learn* OR guid* OR facilitat*)	5308326
#9	TS=(telecommunications OR telemedicine OR tele medicine OR telehealth OR tele health OR tele-health OR ehealth OR mhealth OR m-health OR mobile health)	160853
#10	TS=(ICT)	54137
#11	TS=((inform* OR communicat* OR interact*) NEAR/6 (computer* OR technolog* OR software))	360561
#12	TS=((health* OR treat* OR therap* OR intervention* OR assist* OR selfmanag* OR self-manag*) NEAR/6 (computer* OR technolog* OR software))	233997
#13	TS=(interactive voice response OR IVR)	4619
#14	TS=(internet* OR world wide web OR www OR web OR web-based OR email* OR e-mail* OR online)	1460955
#15	TS=(telephone* OR phone* OR mobile* OR cellphone* OR app OR apps OR text* OR SMS OR smartphone*)	1705579
#16	TS=(virtual reality OR augmented reality OR VR OR AR)	300582
#17	TS=(lay-led OR peer-led OR professionally-led OR therapist-led OR physiotherapist-led OR rheumatologist-led)	2912
#18	#17 OR #16 OR #15 OR #14 OR #13 OR #12 OR #11 OR #10 OR #9 OR #8 OR #7 OR #6 OR #5 OR #4	8748927
#19	#18 AND #3	5655
#20	TS=(exerci* NEAR/2 (train* OR educat* OR teach* OR instruct* OR inform* OR counsel* OR empower* OR advic* OR advis*))	45628
#21	TS=(self NEAR/2 (hydrotherapy))	2
#22	TS=((pain management) NEAR/2 (Train* OR educat* OR teach* OR instruct* OR inform* OR counsel* OR empower* OR advic* OR advis*))	18638

#23	TS=(Cognitive Therapy)	104009
#24	TS=(fatigue NEAR/2 (train* OR educat* OR teach* OR instruct* OR inform* OR counsel* OR empower* OR advic* OR advis*))	2103
#25	TS=((nutrition OR food OR diet) NEAR/2 (train* OR educat* OR teach* OR instruct* OR inform* OR counsel* OR empower* OR advic* OR advis*))	32399
#26	TS=(sleep NEAR/2 (train* OR educat* OR teach* OR instruct* OR inform* OR counsel* OR empower* OR advic* OR advis*))	4501
#27	TS=(Physical Therapy Specialty OR Exercise Therapy)	62498
#28	TS=((Chronic Disease self-management program) OR (CDSMP) OR (Stanford model AND Chronic Disease))	3175
#29	#28 OR #27 OR #26 OR #25 OR #24 OR #23 OR #22 OR #21 OR #20	256479
#30	#29 AND #19	1337
#31	TS=(Qualitative Research OR Interviews as topic OR focus groups OR narration OR questionnaire OR self-report OR attitudes OR tape recording OR Nursing methodology research)	2059691
#32	TS=(qualitative OR ethno* OR emic OR etic OR phenomenolog* OR hermeneutic* OR Heidegger* OR Husserl* OR Colazzi* OR Giorgi* OR Glaser* OR Strauss* OR Van Kaam* OR Van Manen*)	997214
#33	TS=(constant compar* OR focus group* OR grounded theory OR narrative analysis OR lived experience* OR life experience* OR theoretical sampl* OR purposive sampl* OR ricoeur* OR speigelberg* OR merleau* OR metasynthes* or meta-synthes* OR metasummar* OR meta-summar* OR metastud* OR meta-stud* OR maximum variation OR snowball* OR field stud* OR field note* OR fieldnote* OR field record* OR content analy* OR unstructured categor* OR structured categor* OR action research OR audiorecord* OR taperecord* OR videorecord* OR videotap* OR digitalrecord* OR digitaltap*)	4719491
#34	TS=(thematic* NEAR/3 analy*)	82718
#35	TS=((participant* OR nonparticipant* OR non-participant*) NEAR/3 (observ*))	33139
#36	TS=((audio OR tape OR tapes OR taping OR video* OR digital*) NEAR/5 (record* OR interview*))	81192
#37	TS=(interview)	749580
#38	#37 OR #36 OR #35 OR #34 OR #33 OR #32 OR #31	6985108
#39	#38 AND #30	712

Appendix D. Characteristics of the multicomponent self-management interventions explored

Author and year	*Intervention category: Psychological*	*Intervention category: Mind-body therapies*	*Intervention category: Physical activity*	*Intervention category: Lifestyle*	*Intervention category: Medical education*
Bremander 2009	Y	-	Y	-	Y
Feldthusen 2022	Y	-	Y	-	Y
Goksor 2022	Y	-	Y	Y	Y
Gustafsson 2004	Y	-	Y	Y	Y
Mannerkorpi 2003	-	-	Y	-	Y
Bee 2016	Y	-	Y	-	-
Nizza 2018	Y	Y	?	Y	?
Pearson 2020	Y	?	Y	Y	Y
Pearson 2022	?	?	Y	Y	Y
Mengshoel 2021	Y	-	Y	Y	Y
Singstad 2020	Y	Y	-	-	-
Bourgault 2015	Y	-	Y	Y	Y
Lagueux 2021	Y	Y	-	Y	Y
Costa Guedes Miranda 2016	Y	-	-	Y	Y
Oliveira 2019	Y	-	Y	Y	Y
Arfuch 2021	Y	-	Y	-	Y
Rasmussen 2017	Y	-	Y	Y	Y
Mengshoel 2021	Y	-	Y	-	Y
Misje 2023	Y	Y	Y	-	-
Arfuch 2022	Y	-	Y	-	Y
McIlroy 2022	Y	Y	Y	Y	Y
Sharma 2022	Y	-	Y	-	Y
Courel-Ibáñez 2023	Y	-	Y	-	Y
